# Assessing and improving public mental health literacy concerning rTMS

**DOI:** 10.1186/s12888-022-03880-9

**Published:** 2022-04-08

**Authors:** Amanda S. Morrison, Andero Uusberg, Julia Ryan, Amit Goldenberg, Amit Etkin, James J. Gross

**Affiliations:** 1 Department of Psychology, California State University, East Bay, 25800 Carlos Bee Blvd, Hayward, CA 94542 USA; 2 Department of Psychology, Stanford University, Building 420, 450 Jane Stanford Way, Stanford, CA 94305 USA; 3 Harvard Business School, Harvard University, Boston, MA 02163 USA; 4 Department of Psychiatry and Behavioral Sciences, Stanford University, Stanford, CA 94305 USA; 5 Wu Tsai Neurosciences Institute, Stanford University, Stanford, CA 94305 USA; 6 Alto Neuroscience, Los Altos, CA 94022 USA

**Keywords:** Repetitive transcranial magnetic stimulation, Depression, Mental health literacy, Public perception, Neurostimulation, Beliefs

## Abstract

**Background:**

Repetitive transcranial magnetic stimulation (rTMS) has received empirical support as a viable treatment alternative for treatment-resistant major depressive disorder. Nevertheless, patients and the public-at-large may be hesitant to adopt rTMS. In three studies, we sought to (1) assess and (2) improve public perceptions of rTMS as a treatment for depression.

**Methods:**

In Study 1 (*N* = 107), we administered questionnaires on Amazon’s Mechanical Turk (MTurk) to individuals from the US and Canada in a cross-sectional design to assess perceptions of rTMS compared to psychopharmacology, electroconvulsive therapy (ECT), and talk therapy. In Study 2 (*N* = 106), we again used an MTurk sample and a cross-sectional design to assess perceptions of rTMS after providing participants with a relatively long description of rTMS. In Study 3 (*N* = 308), we conducted an experiment in undergraduate students. Participants were randomized to one of four experimental conditions manipulating participants’ understanding of the causal mechanisms of depression prior to assessing their perceptions of rTMS.

**Results:**

Public perceptions of rTMS were more negative than pharmacotherapy and talk therapy but not ECT (Study 1). rTMS perceptions were notably better when participants were given thorough information about rTMS procedures, pain, and side-effects (Study 2), compared to the previous study when they were given a very brief description of rTMS. Finally, perceptions of rTMS were significantly better when participants were given a brain circuitry-based causal explanation of depression compared to when they were given a psychological explanation of the causes of depression (Study 3).

**Conclusions:**

Public perceptions of rTMS are relatively poor. To improve rTMS acceptability, practitioners should carefully consider patients’ prior attitudes and beliefs when explaining rTMS as a treatment alternative. Given that beliefs can have powerful effects on treatment outcome (e.g., placebo, nocebo), future research should explore whether rTMS effects on depression can be improved by facilitating less negative perceptions of rTMS.

**Supplementary Information:**

The online version contains supplementary material available at 10.1186/s12888-022-03880-9.

Although major depressive disorder is a highly prevalent mental disorder with significant morbidity, public mental health literacy about depression, or valid beliefs about the disorder and its treatment, is relatively poor [1, 2]. Only 58% of US adults recognized depression in a child described in a vignette and of these, 12.8% rejected the notion that the problems described were a “mental illness” [3]. When depression is not recognized, this may delay help-seeking. Indeed, data from the World Health Organization’s World Mental Health Surveys showed that of people affected by a mood disorder, the median delay of onset of treatment for those who received treatment ranged from 1 to 14 years [4]. One unfortunate consequence of delayed treatment for depression is that treatment outcome tends to be worse the longer the duration of the depressive episode [5]. In the case of treatment-resistant depression (TRD), when the patient with depression shows insufficient clinical benefit to at least one antidepressant medication trial, the negative consequences of delaying treatment may be compounded.

Beliefs about depression not only shape who seeks treatment, but also how treatments are received. In medicine, perhaps the best-studied example of the impact of beliefs is the placebo effect, which refers to a positive response to a pharmacologically inactive treatment, due in large part to the patient’s expectations of improvement [6]. Unfortunately, expectations are not always positive, and a nocebo effect is said to occur when the patient’s negative expectations lead to a negative response to an inert treatment. With regard to depression treatment, meta-analytic results suggest a significant nocebo response to the expectation of taking antidepressants. Almost half of placebo-treated patients in randomized clinical trials experience at least one drug-related adverse event and approximately 1 in 20 discontinue treatment due to adverse events [7]. The implication of nocebo findings for real treatments is that some adverse reactions, treatment drop-out, and poor response may be due in part to erroneous beliefs about the treatment.

The knowledge that beliefs can have an important impact on treatment response suggests that monitoring and shaping such beliefs is an important aspect of developing novel treatments for depression. One new treatment for TRD which has received increasing empirical attention due to its efficacy, ease of use, and safety is repetitive transcranial magnetic stimulation (rTMS) [8–10]. Unfortunately, it is not yet known how patients and lay people perceive rTMS and similar neurostimulation treatments compared to other treatments. For this reason, efforts are needed to understand how stakeholders perceive innovative, technology-based interventions for depression and how any negative biases that may exist may be corrected.

The one study which has been conducted to-date on perceptions of rTMS in the United States has revealed that even psychiatrists are unfamiliar with how to prescribe rTMS [11]. If providers are unaware of the treatment, patients and the public-at-large are likely also unaware. Moreover, given that rTMS sounds, at face-value, somewhat similar to ECT, which has experienced negative publicity in mass media [12], the public may hold quite negative perceptions of rTMS despite being unfamiliar with it. If this is the case, then patients with depression may shy away from rTMS or be discouraged by loved ones from trying it.

In three studies, we sought to (1) assess public perceptions of rTMS as a depression treatment (Study 1), and (2) attempt to improve perceptions of rTMS (Studies 2, 3). We examined self-reported perceived efficacy of rTMS, likelihood of pursuing rTMS oneself, and likelihood of recommending rTMS to a loved one. All study participants provided written informed consent after receiving a complete description of the respective study and before beginning the study. Each study’s methods and results are presented here in brief and additional details of these methods and results, as well as descriptions of several self-report questionnaires not analyzed for this manuscript, are available in the Supplemental Materials (SM) document accompanying this manuscript. All procedures contributing to this work comply with the ethical standards of the relevant national and institutional committees on human experimentation and with the Helsinki Declaration of 1975, as revised in 2008. All procedures using human subjects were approved by the relevant review body (Studies 1 and 2 by the Stanford University IRB (#7273), Study 3 by the California State University, East Bay IRB (#CSUEB-IRB-2017–200-F)).

## Study 1

We first sought to examine how perceptions of rTMS as a treatment for depression compare to those of other treatments, including pharmacotherapy, electroconvulsive therapy (ECT), and talk therapy. We expected rTMS to be perceived poorly by the public at least in part because of a conflation of rTMS with ECT due to shared superficial features (e.g., stimulation of the brain). Although perceptions of the safety and efficacy of ECT have evolved within the psychiatric community along with the treatment itself, public perceptions have lagged [12].

## Methods

### Participants

Participants were 107 individuals from the U.S. and Canada recruited from Amazon’s Mechanical Turk (MTurk) [13]. See Table 1 for demographic information. All recruited participants responded accurately to an attention check question. Participants were compensated $1.00.

**Table 1:** Participant demographics

	Study 1	Study 2	Study 3
*N*	107	106	308
Age *M*(SD)	33.0(10.2)	32.2(10.0)	20.0(3.3)
% Female	38.3	51.9	53.6
Years of Education *M*(SD)	15.9(2.0)	15.9(2.2)	^a^
% Race/Ethnicity			
African-American/Black	5.6	6.6	9.4
American Indian/Alaskan Native	0.0	0.9	0.6
Asian	10.3	10.4	30.5
Caucasian/White	71.0	76.4	12.0
Latino/Hispanic	9.3	3.8	35.1
Native Hawaiian/Pacific Islander	0.0	0.0	2.6
More than one	3.7	1.9	4.2
Other	-	-	5.5
Unknown/Not reported	0.0	0.0	0.0
% Marital Status			
Single, never married	58.9	55.7	93.2
Married	21.5	27.4	1.3
Living common-law	0.9	0.0	0.3
Living with partner	13.1	9.4	2.6
Divorced	3.7	4.7	0.6
Separated	0.0	0.9	0.0
Widowed	1.9	0.9	0.0
Not reported/Other	0.0	0.9	1.9

*Note*. Studies 1 and 2 were conducted on Amazon’s Mechanical Turk; Study 3 was conducted in an undergraduate sample from a state university on the US West Coast; ^a^Years of education was not assessed, but rather education level and this is reported in the Study 3 Participants section

### Procedure and materials

Using the web-based survey platform Qualtrics to deploy the study online, participants were first presented with a two-sentence description of depression, followed by two-sentence descriptions of each of the four treatments (pharmacotherapy, talk therapy, rTMS, and ECT) one at a time. Each treatment description was followed by five items used to assess *familiarity* and perceptions of rTMS (*likelihood of positive effects*, *likelihood of negative effects*, *likelihood of pursuing*, *likelihood of recommending*). Each question was assessed on an 11-point Likert scale, ranging from 0 “None/Not at all” to 5 “Moderately” to 10 “Extremely.” Next, participants were presented with seven open-ended questions about rTMS. A single close-ended item was then presented to ask about perceived similarity of rTMS to ECT, rated on the same 11-point scale. Following this, participants completed a demographics questionnaire.

### Coding methods

For the open-ended questions, we coded as 1 any description of rTMS in which there was mention of “shock” or related words, or where there was a clear attempt to describe ECT (e.g., “Makes me think about those electronic machines you saw in those old phsychiatric [sic] hospitals long ago that are no longer in service”). All other responses were coded 0.

## Results and Discussion

**Fig. 1:**
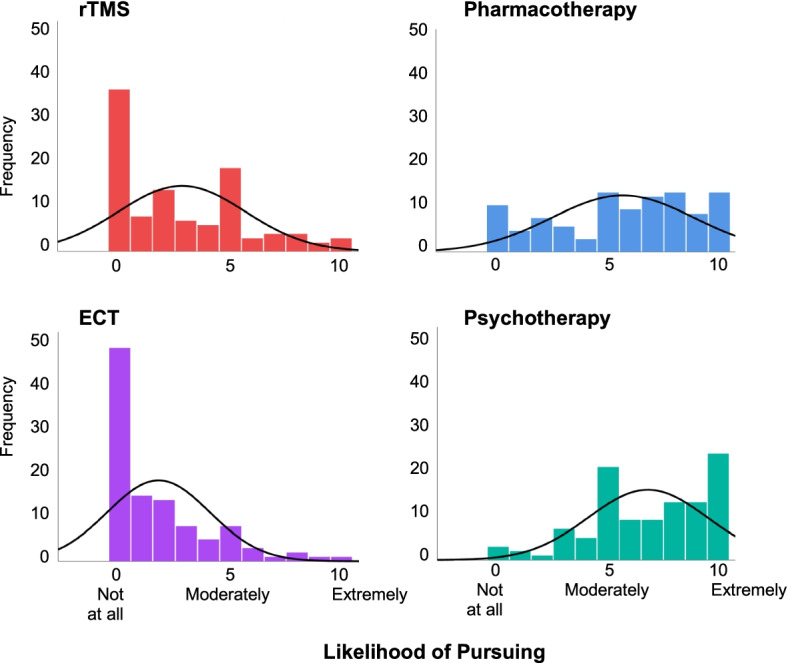
Study 1 Likelihood of Pursuing rTMS and Three Other Treatments *Note*. This figure depicts histograms of participants’ (*N* = 107) ratings on their likelihood of pursuing each of the four treatments from Study 1. Ratings for likelihood of positive effects and likelihood of recommending exhibited the same pattern, with ECT rated the lowest, followed by rTMS, followed by pharmacotherapy, followed by psychotherapy

We first compared familiarity and perceptions of rTMS to the remaining three treatments. rTMS was rated as less familiar than pharmacotherapy, talk therapy, and ECT (*p*s < 0.001; Cohen’s *d* > 0.50). Perceptions of the four treatments also differed (see Fig. 1). Participants viewed ECT in a more negative light than rTMS, but effect sizes indicated that perceptions of rTMS were most often more similar to ECT than to pharmacotherapy or talk therapy. Specifically, three of the perception items (likelihood of positive effects, likelihood of pursuing, and likelihood of recommending) exhibited the same pattern of results from lowest to highest: ECT, rTMS, pharmacotherapy, and talk therapy (rTMS vs. ECT *p*s < 0.009, Cohen’s d*s* > 0.25; rTMS vs. pharmacotherapy *p*s < 0.001, Cohen’s d*s* > 0.80; rTMS vs. talk therapy *p*s < 0.001, Cohen’s d*s* > 0.99). For likelihood of negative effects, ECT was given the highest ratings, followed by rTMS and pharmacotherapy which did not differ from each other, followed by talk therapy (rTMS vs. ECT *p* < 0.001, Cohen’s d = 0.49; rTMS vs. pharmacotherapy *p* = 0.56, Cohen’s *d* = 0.06; rTMS vs. talk therapy *p* < 0.001, Cohen’s d = 0.87).

In response to open-ended questions, 17.8% of participants (*n* = 19) described rTMS in terms that closely matched ECT, even though by this point in the study procedure, all participants had already been provided descriptions of both rTMS and ECT, and the rTMS description did not mention anything about electricity. In response to the final close-ended question which asked participants to rate the similarity of rTMS to ECT, participants rated the two treatments as moderately similar (scale range of 0–10: *M* = 4.94, *SD* = 2.38, 95% CI: 4.49–5.40).

## Study 2

Given that Study 1 used a very brief description of rTMS to educate participants and elicit their judgments, we reasoned that more information about rTMS may be needed to perceive it more positively. Specifically, because rTMS is a relatively unfamiliar treatment, without more detailed information about its procedures, safety, and side effects, then participants’ perceptions may be unfairly biased against the treatment, perhaps because they assume rTMS is similar to the better known, but negatively perceived, ECT. Therefore, in Study 2 we provided participants with the type of information about rTMS that would be provided to a prospective patient, such as information about the procedure itself, the side effects, and more details about the putative mechanisms. We then probed their perceptions with the same questions asked in Study 1.

## Methods

### Participants

Participants were 106 adults from the U.S. and Canada recruited from MTurk who had not participated in Study 1. See Table 1 for demographic information. A total of 108 participants were recruited, but two were dropped for inaccurate response to an attention check item. Participants were compensated $1.00.

## Procedure and materials

Participation again occurred fully online through Qualtrics. Participants were first instructed that we were interested in their personal beliefs and opinions about rTMS as a treatment for depression. They were then shown the same brief definition of depression used in Study 1, followed by extended information about rTMS, including the basics of rTMS, the history of its development, its putative mechanisms in the brain, what the patient experiences, and possible side effects and risks. On a new screen, participants were then presented with five close-ended items assessing *familiarity* and perceptions (*likelihood of positive effects*, *likelihood of negative effects*, *likelihood of pursuing*, *likelihood of recommending*) of rTMS. Then six of the seven open-ended questions from Study 1 were presented. Then a single close-ended item was presented to assess perceived similarity of rTMS to ECT. Participant demographics were assessed last.

### Coding methods

To compare perceptions of rTMS in the current study, in which rTMS was described at length, to perceptions of rTMS in Study 1, in which rTMS was described succinctly, we coded responses to five matching items in the current study and Study 1. Responses to item 1 (general thoughts about rTMS) and item 2 (factors that contributed to perceptions of rTMS) were categorized as negative, neutral/equivocal, or positive. Responses to item 5 (perceived side effects of rTMS) were coded for mention of effects or side effects commonly or historically observed in ECT, with a dichotomous rating of 1 for present (i.e., mention of seizures, brain damage, cognitive evidence of physical brain damage such as memory loss, death) or 0 for absent (i.e., all other responses including typical side effects such as headaches or responses of no side effects). Responses to item 6 (perceptions of pain involved in the procedure) were coded into three categories for degree of pain (no pain, mild discomfort/equivocal, or definite pain). Responses to item 7 (perceptions of rTMS as an appropriate treatment for mild versus severe depression) were coded into three categories for perceived appropriateness for mild depression (yes/no/unsure) and for perceived appropriateness for severe depression (yes/no/unsure).

## Results and Discussion

**Fig. 2:**
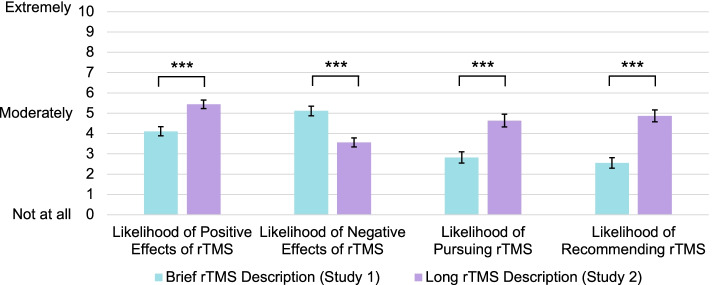
Perceptions of rTMS following Brief and Long Descriptions of rTMS (Studies 1 and 2)**.** *Note*. This figure depicts mean ratings on the four rTMS perception items in Studies 1 (*N* = 107) and 2 (*N* = 106), in which a brief and long description of rTMS were displayed to participants, respectively. Perceptions of rTMS were better following the long description for all four items (*ps* < .001, Cohen’s ds > 0.59). Error bars are standard errors

All four close-ended items assessing perceptions of rTMS indicated that the longer rTMS description yielded superior perceptions than the brief rTMS description. See Fig. 2. Perceptions of the likelihood of positive and negative effects of rTMS, likelihood of pursuing rTMS, and likelihood of recommending rTMS were each rated significantly better in participants who were given the long description in the present Study 2 compared to participants who were given the short description in Study 1 (*p*s < 0.001, Cohen’s *d*s > 0.59). The difference between studies on rTMS familiarity was marginally significant (*p* = 0.07, Cohen’s *d* = 0.25), suggesting that amount of information about rTMS may increase perceived familiarity with it, though the effect size was small. In contrast, participants’ perceived similarity of rTMS to ECT as assessed with a single close-ended question did not differ across the two studies (*p* = 0.51, Cohen’s *d* = 0.09), suggesting that providing a longer rTMS description does not help to differentiate it from ECT.

We also compared responses to the open-ended items in the two studies. When asked what they generally thought about rTMS (item 1), the ratio of the number of participants who provided a positive to neutral to negative response was more positive with a long rTMS description in the present Study 2 (38:35:33) compared to a brief rTMS description in Study 1 (22:38:47), *χ*^2^ (2, *N* = 213) = 6.84, *p* = 0.03, Cramer’s *V* = 0.18. When asked what factors they considered in evaluating their perceptions of rTMS (item 2), there was again a more positive ratio of positive to neutral to negative in the present study (44:29:33) than in Study 1 (17:50:40), *χ*^2^ (2, *N* = 213) = 18.20, *p* < 0.001, Cramer’s *V* = 0.29. When assessing perceived side effects of rTMS (item 5), the same pattern was revealed with the ratio of participants in the current study reporting a more positive perception ratio of non-ECT-like versus ECT-like side effects (98:8), compared to participants in Study 1 (80:27), *χ*^2^ (1, *N* = 213) = 12.13, *p* < 0.001, Cramer’s *V* = 0.24. When assessing perceived pain of rTMS (item 6), again there was a more positive perception ratio of non/mild to moderate to severe in the present study (71:25:10) compared to Study 1 (47:32:28), *χ*^2^ (2, *N* = 213) = 14.26, *p* < 0.001, Cramer’s *V* = 0.26. Finally, when assessing whether rTMS may be an appropriate treatment for mild versus severe depression, participants in the current study were more likely to perceive rTMS as appropriate versus not appropriate versus having no opinion for the treatment of both mild depression (48:46:12) and severe depression (79:17:10) than participants in Study 1 (for mild depression: 27:68:12, *χ*^2^ (2, *N* = 213) = 10.12, *p* < 0.01, Cramer’s *V* = 0.22; for severe depression: 63:32:12, *χ*^2^ (2, *N* = 213) = 6.57, *p* = 0.04, Cramer’s *V* = 0.18.

In conclusion, compared to participants who were given very limited information about rTMS (Study 1), participants in the present study who were given more information about rTMS, perceived rTMS more positively across several measures. Perceived familiarity of rTMS was negligibly higher with a longer rTMS description and perceived similarity of rTMS to ECT did not differ across studies, suggesting observed differences on other ratings were specific to the content provided rather than due to another factor (e.g., history threats). Nevertheless, one significant limitation of the current study is that comparison of a long versus brief rTMS description was conducted across two studies, rather than within one experimental design. Therefore, inferences about causality should be considered cautiously and replication within one experiment with randomization is needed.

## Study 3

In Study 2, we found that longer descriptions of rTMS, similar to what a patient might learn in the process of considering rTMS, were associated with significantly better perceptions of rTMS. However, perceptions of rTMS in Study 2 continued to be relatively negative (see Fig. 2). Therefore, we next sought to examine whether we could further improve rTMS perceptions. Although perceptions of rTMS are likely affected by myriad factors, ranging from the individual to societal level, we chose to focus on a factor which might be easily addressed in a clinical setting. Specifically, we considered whether rTMS perceptions might be adjusted by the framing of the “problem” (i.e., depression). We reasoned that perceptions of causal mechanisms of depression might affect perceptions of the “solution” (i.e., treatment) for depression. Evidence for this reasoning comes from an experiment in which participants who were led to believe their depression was caused by a chemical imbalance subsequently viewed pharmacotherapy as more credible and effective than talk therapy, whereas control participants rated the treatments as equally credible and effective [14].

We randomized participants into one of four conditions which presented different descriptions of depression. In the control condition, participants read about the symptoms of depression. In the remaining three conditions, participants read about a putative cause of depression: brain circuitry-based, neurotransmitter-based, or psychologically-based. We then assessed perceptions of rTMS, pharmacotherapy, and talk therapy, whose described mechanisms matched one of the previously-presented causal explanations, respectively. We expected that descriptions of the “problem” which more closely matched descriptions of the “solution” would result in better perceptions. Therefore, we expected that those assigned to the brain circuitry-based depression description condition would report the most positive perceptions of rTMS, followed closely by the neurotransmitter-based condition, followed by the psychologically-based and control conditions, the latter two of which would not differ from one another.

## Methods

### Participants

Participants were 308 undergraduate students from a state university on the West Coast. Participants were enrolled in an introductory psychology course and received partial course credit as compensation. See Table 1 for demographic information. A total of 319 students were recruited, but 11 answered incorrectly to an attention check question during the study so they were dropped from further analysis. Men were over-recruited so the gender distribution would be more equal (men: *n* = 141, 45.8%; women: *n* = 165, 53.6%). Two individuals identified as “other” gender (0.6%). The sample was highly racially/ethnically diverse, reflecting the composition of the university. Education level was, unsurprisingly, not diverse, with 262 (85.1%) reporting their highest level of education achieved as “some college/vocational school,” 19 (6.2%) as Associate’s degree, and 27 (8.8%) as Bachelor’s degree.

### Procedure and materials

Participation occurred in person in a psychology laboratory. All study materials were presented on computer through Qualtrics. To begin, a research assistant started the study on Qualtrics and left the participant alone in a computer room to complete the study. Participants were not permitted to click backwards in the Qualtrics platform. Within Qualtrics, participants were first randomly assigned to read one of the four depression descriptions that varied on the framing of the putative cause of depression: brain circuitry-based, neurotransmitter-based, psychologically-based, or control (symptom description). Participants were then presented, in random order, three relatively long treatment descriptions (rTMS, pharmacotherapy, talk therapy), one-by-one, each of which was immediately followed by an item assessing *familiarity* and four items assessing perceptions of the treatment (*likelihood of positive effects*, *likelihood of negative effects*, *likelihood of pursuing*, *likelihood of recommending*). Participants then completed a demographics questionnaire.

## Results and Discussion

**Fig. 3:**
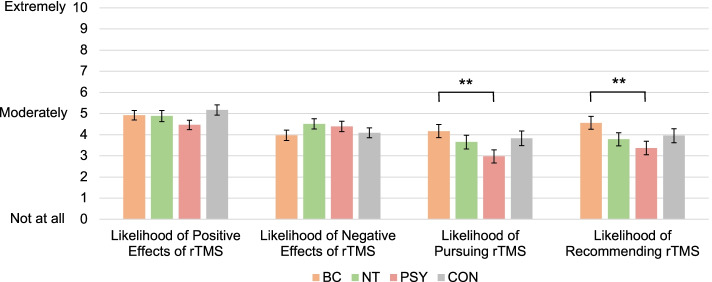
Study 3 Perceptions of rTMS by Depression Description Condition**.** *Note.* This figure depicts mean ratings on the four rTMS perception items by experimental condition. Participants in the BC condition rated likelihood of pursuing and recommending rTMS significantly higher than the PSY condition (*p*s < .009, Cohen’s *d*s = 0.44). Error bars are standard errors. BC = Brain circuitry-based causal description (*n* = 78); NT = Neurotransmitter-based causal description (*n* = 77); PSY = Psychologically-based causal description (*n* = 76); CON = Control (symptom) description (*n* = 77)

After we confirmed that there was no evidence of failure of random assignment to the four conditions, we compared the four conditions on each of the four rTMS perception items using omnibus analyses of variance followed by independent samples *t*-tests as warranted (see Fig. 3). Omnibus tests for likelihood of pursuing and likelihood of recommending rTMS approached statistical significance (*p*s = 0.067, 0.062; ${\eta }_{p}^{2}$ s = 0.02). Follow-up pairwise comparisons revealed that, consistent with our hypothesis, rTMS was viewed more favorably among those who were randomized to read a brain circuitry-based causal description of depression (the putative closest match to the mechanisms of rTMS) compared to those who read a psychologically-based causal description of depression (the putatively furthest match to the mechanisms of rTMS) (*p*s < 0.009, Cohen’s *d*s = 0.44). No other tests were significant. Therefore, contrary to our hypothesis, the neurotransmitter-based and control (symptom) descriptions of depression were not significantly inferior to the brain circuitry-based description, nor were they significantly superior to a psychologically-based description.

## General discussion

There is growing evidence that rTMS is a relatively safe and effective psychiatric treatment for TRD [8–10], but we know little about how the general public and potential patients perceive rTMS. To address this gap in our knowledge, we conducted a series of three studies in which we examined how the general public perceives rTMS relative to other mental health treatments (Study 1) and whether negatively biased perceptions of rTMS could be modified (Studies 2, 3).

We found rTMS to be perceived more poorly than other treatments for depression. Participants rated that they perceived rTMS to have a lower likelihood of positive effects and were less willing to pursue or recommend rTMS compared to pharmacotherapy and talk therapy. The one treatment which was perceived slightly worse than rTMS was ECT, and people also perceived rTMS to have similar likelihood of negative effects as pharmacotherapy. Poor perceptions of rTMS did not seem to be due to lack of familiarity with rTMS, as rTMS was rated as less familiar yet more positive than ECT (Study 1). Rather, poor perceptions of rTMS may be partially due to conflating rTMS with ECT. When given the opportunity to answer open-ended questions about rTMS (Study 2), a sizable proportion of participants provided incorrect descriptions of rTMS that actually described ECT (18%) and described (side) effects more closely aligned to ECT than to rTMS, such as seizures and memory loss (25%).

Although perceptions of rTMS in the general public appear to be rather negative (Study 1), we also found evidence that these beliefs could be modified. Perceptions of rTMS were significantly better when participants were provided detailed information about rTMS, such as what the patient experiences, common side effects, and putative mechanisms (Study 2). In addition, perceptions of rTMS were significantly better when depression was described as a problem which arises from impaired communication in brain circuits compared to when depression was described as being caused by problems with thinking and behavior (Study 3).

### Limitations and directions for future research

There are several limitations to the current set of studies. All study results are based on self-report, so future research should examine people’s behavior toward rTMS. Likewise, all participants were from the general public and future research should examine how treatment-seeking individuals perceive rTMS. With regard to sample characteristics, participant demographics in Studies 1 and 2 (MTurk) differed from those in Study 3 (undergraduates). Specifically, Study 3 participants were younger on average, more racially/ethnically diverse, more likely to be single, and slightly less educated, though more homogenous in their highest level of education. Although perceptions of rTMS were similar in Study 3 compared to Studies 1 and 2, and perceptions of rTMS were consistently more negative than perceptions of pharmacotherapy and talk therapy in both Studies 1 and 3 (see SM Study 3 Results), replication of Study 3 findings in an older, more educationally heterogeneous sample is important for generalizability. Although we observed some statistically significant improvements in rTMS perceptions based on modifying the description of depression, the effect sizes of these findings were small-to-moderate, suggesting that further research is needed to address the negatively biased beliefs that people have toward rTMS. Finally, further research is needed to fully understand the myriad factors that contribute to poor perceptions of rTMS. For example, people may not understand the temporary nature of a single TMS pulse – many participants responded consistent with a belief that the brain would suffer trauma due to magnetic stimulation. People’s perceptions may be improved by viewing images or video of actual rTMS recipients, or perhaps learning more about magnetic stimulation itself. 

## Conclusions

Understanding how potential stakeholders perceive rTMS as a treatment for mental illness is important for several reasons. Many patients with depression do not respond to pharmacotherapy and rTMS is a viable alternative that is relatively safe. If patients are unwilling to try rTMS or if they experience nocebo effects to rTMS treatment due to negative beliefs about rTMS, then they will not have the opportunity to benefit from the treatment.

## Supplementary Information

**Additional file 1.**

## Data Availability

Each study’s methods and results are presented in this manuscript in brief and further details are provided in the supplementary online materials document accompanying this manuscript. Additional details of these methods and results, as well as descriptions of several self-report questionnaires not analyzed for this manuscript and all data, are available from the corresponding author upon reasonable request.
