# Supplementary material for: Assessing and improving public mental health literacy concerning rTMS
Source: BMC Psychiatry. 2022 Apr 8;22:249. doi: 10.1186/s12888-022-03880-9 (PMC8991954; doi:10.1186/s12888-022-03880-9)
Supplement: Supplementary file 1 — Additional file 1. [file 12888_2022_3880_MOESM1_ESM.docx]

**Supplemental Online Materials**

Supplement for Morrison, Uusberg, Ryan, Goldenberg, Etkin, & Gross. Assessing and Improving Mental Health Literacy Concerning rTMS.

Table of Contents

[I. Study 1 supplemental Method and Results sections 2](#_Toc46411318)

[II. Study 2 supplemental Method and Results sections 8](#_Toc46411319)

[III. Study 3 supplemental Method and Results sections 13](#_Toc46411320)

[IV. References 25](#_Toc46411321)

#

# Study 1 supplemental Method and Results sections

**Study 1 Supplemental Methods and Materials**

**Procedure**

Order of presentation of the four treatments (rTMS, ECT, pharmacotherapy, talk therapy) was randomized across participants. At the end of the study, at the end of the demographics questionnaire, two items were presented that asked participants about personal experience with mental illness (for further information, see Materials and Results sections below).

**Materials**

**Depression and Treatment Descriptions**

The depression description and descriptions of all four treatments were two sentences long. The treatment descriptions were written to approximate one another in terms of length, content, and tone.

***Depression Description***

“Depression is a condition characterized by low or sad mood and a loss of interest in normal activities that lasts most of the day, continuously for at least 2 weeks. Depression may also be associated with fatigue, feelings of worthlessness or guilt, difficulty concentrating, sleep disruption, thoughts of suicide, or change in appetite.”

***rTMS Description***

“Repetitive transcranial magnetic stimulation is a treatment that involves the patient receiving small pulses from an electromagnet at his/her scalp from a physician to address abnormalities in brain circuitry involved in depression. Repetitive transcranial magnetic stimulation is thought to help depression through enhancing the natural electrical signal pathways that connect different brain regions.”

***ECT Description***

“Electroconvulsive therapy is a treatment that involves the patient receiving, while under general anesthesia, small electric currents through the brain from electrodes placed on the scalp. Electroconvulsive therapy is thought to help depression by inducing brief, controlled seizures, which are known to change many chemical aspects of brain function.”

***Pharmacotherapy Description***

“Pharmacotherapy is a treatment that involves the patient taking medication prescribed by a physician to address brain chemical abnormalities associated with depression. Pharmacotherapy is thought to help depression through balancing the levels of certain neurotransmitters, such as serotonin, in the brain.”

***Talk Therapy Description***

“Talk therapy is a treatment that involves the patient talking with a therapist or counselor to better understand their difficulties and to learn coping skills to manage their depression. Many forms of talk therapy are thought to help depression through teaching the patient to challenge negative styles of thinking and approach and manage, rather than withdraw from, difficult emotions and situations.”

**Familiarity with and Perceptions of Treatment**

Participants rated each of the following five items for each of the four treatments, which included, “repetitive transcranial magnetic stimulation,” “electroconvulsive therapy,” “pharmacotherapy,” and “talk therapy.” Each item was rated on an 11-point scale ranging from 0 “Not at all” to 5 “Moderately” to 10 “Extremely.” *Familiarity* of each treatment was assessed with the question, “How familiar are you with [treatment]?” *Likelihood of positive effects* was assessed with the question, “When someone has depression, how likely do you think it is that [treatment] could have positive effects?” *Likelihood of negative effects* was assessed with the question, “When someone has depression, how likely do you think it is that [treatment] could have negative effects?” *Likelihood of pursuing* was assessed with the question, “If you had depression, how likely would you be to pursue [treatment]?” *Likelihood of recommending* was assessed with the question, “If a loved one had depression, how likely would you be to recommend [treatment]?”

**Open-Ended rTMS Items**

We asked seven open-ended questions about rTMS, which began vague and became progressively more targeted. The first open-ended question (item 1) was “What did you think about repetitive transcranial magnetic stimulation when you were reading the description?” The second (item 2) was “What factors contributed to your decision about how likely you would be to pursue or recommend repetitive transcranial magnetic stimulation?” The third (item 3) was “What treatments for depression would you prefer to repetitive transcranial magnetic stimulation and why?” The fourth (item 4) was “How long do you think repetitive transcranial magnetic stimulation has been around as a treatment?” The fifth (item 5) was “Do you think any side effects are common with repetitive transcranial magnetic stimulation treatment? If so, which?” The sixth (item 6) was “Do you think repetitive transcranial magnetic stimulation sounds like a painful treatment? If yes, how painful?” The seventh and final open-ended question (item 7) was “What are your views on repetitive transcranial magnetic stimulation for treatment of mild versus severe depression? Severe depression includes symptoms such as suicidal thoughts/behavior or psychotic experiences (seeing or hearing things that are not real).”

**Comparing rTMS to ECT**

One close-ended question was included to assess perceived similarity of rTMS to ECT: “How similar do you think repetitive transcranial magnetic stimulation is to electroconvulsive therapy (i.e., “electroshock therapy”)?” The item was rated on an 11-point Likert-type scale, from 0 “Not at all the same” to 5 “Moderately” to 10 “Completely the same.”

**Mental Health Items**

In the demographics questionnaire presented at the end of the study, participants also responded to two questions about mental health experience. The first asked, “Have you or a close loved one ever been diagnosed with a mental illness, including depression?” Participants were provided three response options including, “Yes,” “No,” and “Unsure.” Then participants were asked, “Have you or a close loved one ever sought treatment for a mental illness, including depression?” Again, participants were provided three response options of “Yes,” “No,” and “Unsure.”

**Coding Methods**

We coded four of the seven open-ended items (items 1, 2, 5, and 6) for mention of “shock,” “electroshock,” “electric stimulation,” or “[electrical] current,” or a clear attempt to describe ECT which did not mention any of the previous words (e.g., “Makes me think about those electronic machines you saw in those old phsychiatric [sic] hospitals long ago that are no longer in service”). All other responses to these items were coded 0 (e.g., “It seemed safe and reliable,” “It scares me to alter brain waves in that manner”). We did not code item 3 because it asked participants to identify which treatment(s) they would prefer to rTMS and thus when mentioning words like “shock” could have been referring to ECT or rTMS (e.g., “Nothing with electrical shocks … Talk therapy is about as far as I am willing to go” or “I would prefer talking first… Getting small shocks to the brain make [sic] trick you into feeling better but I don't think it is going to solve what makes you depressed…”). We did not code item 4 (perception of how long rTMS has been in use) because, although some responses may have alluded to ECT (e.g., “100's of years” or “Since the 1800's”), it was not possible to determine whether these responses were clear conflations of rTMS with ECT. We did not code item 7 in the analyses for this study but did for Study 2 (see Study 2 Supplemental Method and Results).

**Study 1 Supplemental Results**

**Sample Description: Mental Health Experience**

In response to whether the participant or a close loved one had ever been diagnosed with a mental illness, more than half reported “Yes” (57.9.6%), one third reported “No” (35.5%), and the remaining participants reported “Unsure” or did not respond (6.5%). In response to the question about whether the participant or a close loved one had ever been treated for a mental illness, again more than half reported “Yes” (56.1%), over one third reported “No” (39.3%), and the remaining participants reported “Unsure” or did not respond (4.7%).

**Familiarity of rTMS versus other treatments**

We first compared familiarity and perceptions of rTMS to the remaining three treatments by conducting repeated measures analyses of variance (ANOVAs), followed by paired samples *t*-tests to probe significant omnibus tests. The four treatments were rated as differentially familiar, *F*(3, 318) = 177.30, *p* < .001, ${}_{p}^{2}$ = .63, with rTMS rated as the least familiar [versus pharmacotherapy: *t*(106) = 17.36, *p* < .001, Cohen’s *d* = 1.68; talk therapy, *t*(106) = 18.01, *p* < .001, Cohen’s *d* = 1.74; ECT, *t*(106) = 5.68, *p* < .001, Cohen’s *d* = 0.55]. ECT was rated as the second least familiar [versus pharmacotherapy: *t*(106) = 11.99, *p* < .001, Cohen’s *d* = 1.16; talk therapy: *t*(106) = 13.09, *p* < .001, Cohen’s *d* = 1.27]. Pharmacotherapy was rated as marginally less familiar than talk therapy, *t*(106) = 1.94, *p* = .05, Cohen’s *d* = 0.19.

Full statistical results for the treatment perception items, reported in the primary manuscript, are provided below in Table S1.

**Table S1**

*Study 1 Statistical Results Comparing Perceptions of rTMS to Other Treatments*

|  | df | *F* or *t* | *p* | ${}_{p}^{2}$ or Cohen’s *d* | df | *F* or *t* | *p* | ${}_{p}^{2}$ or Cohen’s *d* |
| --- | --- | --- | --- | --- | --- | --- | --- | --- |
|  | **Likelihood of positive effects** | | | | **Likelihood of negative effects** | | | |
| Omnibus | 3, 318 | 116.02 | < .001 | .52 | 3, 318 | 69.87 | < .001 | .40 |
| Pairwise |  |  |  |  |  |  |  |  |
| rTMS vs. ECT | 106 | 2.70 | .008 | 0.26 | 106 | 5.05 | < .001 | 0.49 |
| rTMS vs. pharmacotherapy | 106 | 9.68 | < .001 | 0.94 | 106 | 0.59 | .56 | 0.06 |
| rTMS vs. talk therapy | 106 | 12.12 | < .001 | 1.17 | 106 | 9.04 | < .001 | 0.87 |
| Pharmacotherapy vs. talk therapy | 106 | -3.95 | <.001 | 0.38 | 106 | 11.26 | <.001 | 1.09 |
| Pharmacotherapy vs. ECT | 106 | 12.03 | <.001 | 1.16 | 106 | -3.56 | .001 | 0.34 |
| Talk therapy vs. ECT | 106 | 13.92 | <.001 | 1.35 | 106 | -12.08 | <.001 | 1.17 |
|  |  |  |  |  |  |  |  |  |
|  | **Likelihood of pursuing** | | | | **Likelihood of recommending** | | | |
| Omnibus | 3, 318 | 100.10 | < .001 | .49 | 3, 318 | 138.82 | < .001 | .57 |
| Pairwise |  |  |  |  |  |  |  |  |
| rTMS vs. ECT | 106 | 4.81 | < .001 | 0.46 | 106 | 3.94 | < .001 | 0.38 |
| rTMS vs. pharmacotherapy | 106 | 8.42 | < .001 | 0.81 | 106 | 9.37 | < .001 | 0.91 |
| rTMS vs. talk therapy | 106 | 10.31 | < .001 | 1.00 | 106 | 13.53 | < .001 | 1.31 |
| Pharmacotherapy vs. talk therapy | 106 | 3.28 | .001 | 0.32 | 106 | -5.67 | <.001 | 0.55 |
| Pharmacotherapy vs. ECT | 106 | 11.26 | <.001 | 1.09 | 106 | 11.29 | <.001 | 1.09 |
| Talk therapy vs. ECT | 106 | 14.86 | <.001 | 1.44 | 106 | 17.25 | <.001 | 1.67 |

*Note*. Omnibus tests are repeated measures analyses of variance (ANOVA); pairwise tests are paired samples *t*-tests; effect size for omnibus tests is ${}_{p}^{2}$ and for pairwise tests is Cohen’s *d* (mean difference divided by standard deviation of the difference); all tests are two-tailed; rTMS = repetitive transcranial magnetic stimulation; ECT = electroconvulsive therapy.

# Study 2 supplemental Method and Results sections

**Study 2 Supplemental Methods and Materials**

**Procedure**

As in Study 1, at the end of the demographics questionnaire, which was presented last in the study procedure, two items about the participants’ personal mental health experience were presented.

**Materials**

**rTMS Description**

“What is repetitive transcranial magnetic stimulation? Repetitive transcranial magnetic stimulation (rTMS) uses a magnetic coil to stimulate specific brain regions. It is non-invasive and the patient can resume normal activity immediately after stimulation.

rTMS uses an electromagnet that generates magnetic field pulses (that are similar in strength to an MRI scan) which cause small electric currents that stimulate nerve cells in the targeted brain regions. rTMS stimulates a small area on the scalp, about the size of a quarter. A plastic-coated magnetic coil is held against the patient’s head. The patient hears a clicking noise as a few magnetic pulses are produced. These magnetic pulses induce very brief activity in brain areas underlying the TMS coil. Stimulation intensity is calibrated according to the amount of energy needed in the coil to induce activity specifically in each patient’s brain. To do so, the physician increases the intensity of the stimulation until it causes either the patients thumb to move (when the coil is placed over the part of the brain controlling movement on the other side of the body) or causes the patient to see a blob of light (when the coil is placed on the back of the patient’s head). This calibration is done to ensure that sufficient power is used for each individual without excessive stimulation.

During rTMS, the patient receives repetitive pulses either continuously when at low frequency (≤1 Hz or one time per second) or during brief periods lasting several seconds when at >1Hz. Stimulation periods are separated by a rest period, consistent with published TMS safety guidelines. At no point does TMS exceed 60Hz (60 times per second).

rTMS has been studied since the mid-1990’s and has been approved by the FDA as a treatment for major depression. The magnetic pulse of rTMS stimulates nerve cells in the region of the brain involved in mood control and depression, and it may activate regions of the brain that have decreased activity in people with depression. Though the biology of why rTMS works is not completely understood, the stimulation appears to affect how this part of the brain is working, which in turn seems to ease depression symptoms and improve mood. rTMS is typically used when standard treatments such as medications and talk therapy don’t work.

What does the patient experience? rTMS does not involve any anesthesia or sedation, therefore, the patient remains awake and alert during the treatment. The patient sits in a chair and is asked to wear ear plugs to protect his/her hearing since the TMS device emits a clicking noise when the magnetic pulses are produced. The patient may also be asked to wear a swim cap for making measurements of their head. Generally, people feel a slight knocking or tapping on the head as pulses are administered.

A typical rTMS session lasts 30 to 60 minutes. Typically, patients receive multiple treatments, which are delivered 5 days a week for 4 to 6 weeks. Typically, between treatments, the patient can expect to work and drive.

What are the possible side effects/risks? rTMS is considered to be a low-risk procedure. The only common side effect of rTMS (approx. 25% of patients) is a mild headache. The patient may also feel discomfort at the stimulated site because the muscles of the scalp/jaw/face may contract or tingle during the procedure. Brief light-headedness may also occur. There are no known significant risks with this procedure at this time because the magnetic fields at the strengths used are thought to be without harm. The exception is if the patient has a cardiac pacemaker, or a certain type of metallic clip in his/her body (i.e., an aneurysm clip in the brain). In patients with epilepsy, activation of the brain could also activate a seizure. Patients with stroke can also develop seizures due to the brain scar. Therefore, magnetic stimulation of the brain could conceivably activate a seizure in a stroke survivor with such a scar. Therefore, those with a history of epilepsy or stroke are excluded from receiving rTMS. For a normal healthy person, producing a seizure from rTMS is very unlikely.

There are no known long-term adverse effects reported with the use of this device. Rarely, device malfunction could result in a scalp burn. There may be unforeseen risks in the long-term that are currently unknown. The TMS device produces a clicking sound. Although studies have found no hearing impairments as a result of this sound, some patients experience a mild temporary effect on their hearing. To minimize this possibility, patients are given protective earplugs or headphones. Although uncommon, some patients have experienced nausea during the procedure. If this occurs, the patient can discontinue the procedure. Objects such as watches and credit cards are also removed as these could be damaged. If the patient is or could be pregnant, the effects of TMS on a fetus are unknown and, therefore, the procedure is not performed. Patients may also experience temporary and local bruising, swelling, or pain from the swim cap and/or muscle activation by TMS.”

**Open-Ended rTMS Items**

We presented participants with six of the seven items used in Study 1. We excluded item 4 from Study 1, which assessed perceptions of how long rTMS has been around as a treatment, because this specific information was provided in the extended rTMS description used in this study.

**Comparing rTMS to ECT**

The same close-ended question used in Study 1 to assess perceived similarity of rTMS to ECT was included in the current study. The item was rated on an 11-point Likert type scale, ranging from 0 “Not at all” to 5 “Moderately” to 10 “Completely.”

**Mental Health Items**

The same two personal mental health experience items used in Study 1 were presented in this study at the end of the demographics questionnaire. Participants rated their personal experience with mental illness diagnosis (yes/no/unsure) and treatment (yes/no/unsure).

**Coding Methods**

Five of the six items that were presented in both the current study and Study 1 were coded. As in Study 1, we did not code item 3, which assessed preference for treatments other than rTMS, as this cued responses that were largely irrelevant to rTMS.

**Study 2 Supplemental Results**

**Sample Description: Mental Health Experience**

The proportions of participants who reported personal experience with mental illness in the current study were very similar to Study 1. In the current sample, just over half of participants (53.8%) reported that they or a close loved one had ever been diagnosed with a mental illness, less than half reported that they had not (43.4%), and the remaining participants reported “Unsure” or did not respond (1.9%). Likewise, with regard to whether the participant or a close loved one had ever been treated for a mental illness, more than half reported “Yes” (56.6%), less than half reported “No” (40.6%), and the remaining participants reported “Unsure” or did not respond (2.8%).

**Familiarity and Perceptions of rTMS Across Studies 1 and 2**

To examine whether familiarity or perceptions of rTMS were improved with the provision of extended information about rTMS, we first compared responses to the close-ended familiarity and perception ratings of rTMS from the current study with familiarity and perception ratings from Study 1. A significant limitation of this approach is that participants for this comparison came from different studies, so we cannot be confident about inferences of causality. We did not randomize participants to these different conditions in one study. Therefore, the following results should be replicated in a single experimental study.

We conducted a series of independent samples *t*-tests on the rTMS familiarity and perception items with Study (1, 2) serving as the independent variable. Compared to participants who were provided a brief description of rTMS (Study 1), participants who were given the longer description (present Study 2) reported significantly: (1) higher likelihood of positive effects of rTMS, *t*(211) = 4.33, *p* < .001, Cohen’s *d* = 0.60, (2) lower likelihood of negative effects of rTMS, *t*(211) = 4.78, *p* < .001, Cohen’s *d* = 0.66, (3) higher likelihood of pursuing rTMS, *t*(211) = 4.34, *p* < .001, Cohen’s *d* = 0.60, and (4) higher likelihood of recommending rTMS, *t*(211) = 5.92, *p* < .001, Cohen’s *d* = 0.82. The difference between studies on rTMS familiarity was marginally significant, *t*(211) = 1.84, *p* = .07, Cohen’s *d* = 0.25. In contrast, participants’ perceived similarity of rTMS to ECT as assessed with a single close-ended question did not differ across the two studies, *t*(211) = 0.66, *p* = .51, Cohen’s *d* = 0.09.

# Study 3 supplemental Method and Results sections

**Study 3 Supplemental Methods and Materials**

**Procedure**

After reading about and rating all three treatments, participants were presented with several questionnaires. First, they completed brief self-report measures of anxiety and depression. Then they completed three additional questionnaires not analyzed in the current study. The first assessed participants’ beliefs about the malleability of well-being, the second assessed participants’ perceptions of public stigma towards mental illness, and the third assessed participants’ perceptions of their own stigmatizing beliefs toward mental illness. Last, they completed a demographics questionnaire which included the same two items assessing personal mental health experience used in Studies 1 and 2.

**Materials**

**Depression Descriptions**

Each of the four depression descriptions was about two paragraphs in length. We attempted to mimic tone and complexity of material across the four descriptions. The descriptions were revised for clarity based on feedback from undergraduate student research assistants.

***Brain Circuitry-Based Depression Description***

In the brain circuitry-based causal description, participants were provided education about circuits in the brain which connect different regions of the brain. They were told that the circuitry connecting the amygdala and prefrontal cortex are disrupted in those with depression:

“Current research shows that, compared to non-depressed individuals, depressed individuals exhibit disruptions in several brain circuits. Brain circuits are pathways of interconnected neurons, or brain cells. These pathways allow different brain regions to communicate with each other.

One brain circuit involved in regulating or managing our emotions and behaviors is that between the prefrontal cortex and the amygdala. The prefrontal cortex is the outer portion of the front part of our brain. It is responsible for executive function, or our abilities to plan, reason, and control urges and emotions. The amygdala is a small, almond-shaped structure located deep in the center of the brain. It is primarily responsible for our experience of emotions. When these two structures are communicating as they should, we see closely-coupled activation in these two areas, meaning that when one is activated, the other becomes quickly activated.

Brain research has shown that, compared to people who are not depressed, people who are depressed have reduced communication between these two brain areas. When these two regions are not well-connected, the individual may have a difficult time controlling strong negative emotions, such as fear and sadness, or in engaging in the behaviors that they value.”

***Neurotransmitter-Based Depression Description***

In the neurotransmitter-based causal description, participants were provided information about neural communication and about how certain neurotransmitters, such as serotonin and dopamine, are responsible for positive emotions and energy and are lacking in individuals with depression:

“Current research shows that, compared to non-depressed individuals, depressed individuals exhibit disruptions in several neurotransmitter systems. Neurotransmitters are the chemical messengers which communicate between neurons, or brain cells.

Neurotransmitters from one neuron can activate or inhibit a second neuron by crossing the small space between them, called the synapse, and binding to receptors on the second neuron. If the signal is activating, the message gets passed farther along. If the signal is inhibitory, the message is suppressed. Neurotransmitters also affect the neuron from which they came. Once a certain amount of the neurotransmitter has been released into the synapse, feedback is sent to stop sending more neurotransmitter and to start bringing it back into the cell, a process called reuptake. Enzymes break down the remaining neurotransmitters in the synapse.

In people who are depressed, this complex system has gone wrong in some way. For example, receptors may be insensitive to a specific neurotransmitter, causing their response to its release to be inadequate. Or a message might be weakened if the originating cell pumps out too little of a neurotransmitter or an overly efficient reuptake mops up too much before the neurotransmitters have a chance to bind to the second neuron. Any of these disruptions could significantly affect mood. A few neurotransmitters that play a role in depression include acetylcholine, serotonin, norepinephrine, dopamine, and gamma-aminobutyric acid (GABA).”

***Psychologically-Based Depression Description***

In the psychologically-based causal description, participants were told that beliefs contribute to emotions and behaviors, and that depression results from negative thinking styles and withdrawal behaviors:

“Current research shows that, compared to non-depressed individuals, depressed individuals exhibit disruptions in their thinking patterns. The way that we think plays a significant role in how we feel and behave.

Our moment-to-moment thoughts stem from our beliefs, or our acceptance that something is true or that something exists. Beliefs form over the course of our lives, often from our role models and our learning experiences. For example, you may believe that people are generally good if this is what your caregivers taught you and you never encountered a situation that changed your mind. Our beliefs powerfully shape our emotions and our behavior. For example, people who believe that others are generally good tend to be friendly, whereas people who believe that others can’t be trusted tend to be socially withdrawn.

Psychological research has shown that, compared to people who are not depressed, people who are depressed hold negative beliefs about themselves, the world around them, and their future, which, taken together, is called the “negative cognitive triad.” Depressed individuals are more likely to believe, for example, that they are worthless, that others don’t like them, and that matters are unlikely to improve over time. These beliefs contribute to feelings of depression directly, as well as indirectly through their effect on behavior. Holding these beliefs leads to behaviors such as isolating oneself, ignoring positive feedback from others, and withdrawing from challenges.”

***Control Depression Description***

In the control condition, participants were provided a thorough description of the *symptoms* of depression but were not told anything about putative causes of depression:

“Current research shows that, compared to non-depressed individuals, depressed individuals exhibit symptoms that are either significantly distressing or significantly interfering in daily life, or both.

Distress refers to the range of difficult emotions that people with depression experience. The specific types, intensities, and durations of difficult emotions varies across depressed individuals. Most frequently, people with depression experience feelings of sadness, emptiness, or anxiety. Feelings of helplessness, worthlessness, or guilt are also quite common, as are feelings of hopelessness and irritability.

In addition to this distress, depression is associated with a variety of physical symptoms, which can also interfere in daily living. People with depression may feel extremely tired or think more slowly. Daily routines and tasks may seem too hard to manage. For some, it may be difficult to focus. Simple things, like reading a newspaper or watching TV, may be hard. Some people experience difficulties remembering details. It might also seem overwhelming to make a decision, whether it’s big or small. Compared to people who are not depressed, people who are depressed may wake up too early or have insomnia, or sleep longer than usual. They may overeat or not feel hungry. Finally, they may experience headaches, cramps, an upset stomach, or digestive problems.”

**Treatment Descriptions**

The rTMS description was highly similar to that used in Study 2. The descriptions of pharmacotherapy and talk therapy were written to mimic the rTMS description in length, tone, and topics covered. The treatment descriptions were reviewed by undergraduate student research assistants and modified for clarity.

***rTMS Description***

**“Repetitive transcranial magnetic stimulation (rTMS) has been studied since the mid-1990’s and has been approved by the FDA as a treatment for major depression. The magnetic pulse of rTMS stimulates nerve cells in the prefrontal cortex, the region of the brain responsible for** our abilities to plan, reason, and control urges and emotions. The prefrontal cortex communicates with other areas of the brain, such as the amygdala, which is located deep in the center of the brain and is responsible for our experience of emotion. **Though the biology of why rTMS works is not completely understood, the stimulation appears to affect how the prefrontal cortex communicates with other brain areas such as the amygdala.**

What is rTMS? **rTMS uses a magnetic device to stimulate specific brain regions. It is non-invasive and the patient can resume normal activity immediately after stimulation.**

**rTMS uses an electromagnet that generates magnetic field pulses which cause small electric currents. The strength of the magnetic field is like an MRI scan (what physicians use to look at organs and structures inside the body). The small electric currents of the TMS device stimulate nerve cells in a targeted brain region, about the size of a quarter, just underneath the scalp. The TMS device is a plastic-coated magnetic coil which is held against the patient’s head. The patient hears a clicking noise as a few magnetic pulses are produced. Stimulation intensity is attuned to each patient so that sufficient, but not excessive, power is used.**

**During rTMS, the patient receives repetitive pulses either continuously (at the relatively slow rate of one pulse/second), or during brief, separated periods lasting several seconds each (at a relatively faster rate of up to 60 pulses/second).**

**What does the patient experience?** **rTMS does not involve any anesthesia or sedation, therefore, the patient remains awake and alert during the treatment. The patient sits in a chair and is asked to wear ear plugs to protect his/her hearing since the TMS device emits a clicking noise when the magnetic pulses are produced.** **The patient may also be asked to wear a swim cap for making measurements of their head.  Generally, people feel a slight knocking or tapping on the head as pulses are administered.** **A typical rTMS session lasts 30 to 60 minutes. Typically, patients receive multiple treatments, which are delivered 5 days a week for 4 to 6 weeks. Typically, between treatments, the patient can expect to work and drive.**

**What are the possible side effects/risks?  rTMS is considered to be a low-risk procedure.  The only common side effect of rTMS (approx. 25% of patients) is a mild headache.  The patient may also feel discomfort at the stimulated site because the muscles of the scalp/jaw/face may contract or tingle during the procedure. Brief light-headedness may also occur. There are no known significant risks with this procedure at this time because the magnetic fields at the strengths used are thought to be without harm.  The exception is if the patient has a cardiac pacemaker, or a certain type of metallic clip in his/her body (i.e., an aneurysm clip in the brain).  In patients who have had seizures, activation of the brain could also activate a seizure. Patients who have had a stroke can also develop seizures due to the brain scar.** Therefore, magnetic stimulation of the brain could conceivably activate a seizure in a stroke survivor with such a scar.  **Therefore, those with a history of seizures or a stroke are excluded from receiving rTMS. For a normal healthy person, producing a seizure from rTMS is very unlikely.**

**There are no known long-term adverse effects reported with the use of this device.  Rarely, device malfunction could result in a scalp burn.  There may be unforeseen risks in the long-term that are currently unknown.  The TMS device produces a clicking sound.  Although studies have found no hearing impairments because of this sound, some patients experience a mild temporary effect on their hearing.  To minimize this possibility, patients are given protective earplugs or headphones.  Although uncommon, some patients have experienced nausea during the procedure.  If this occurs, the patient can discontinue the procedure.  Objects such as watches and credit cards are also removed as these could be damaged.  If the patient is or could be pregnant, the effects of TMS on a fetus are unknown and, therefore, the procedure is not performed.  Patients may also experience temporary and local bruising, swelling, or pain from the swim cap and/or muscle activation by TMS.”**

***Pharmacotherapy Description***

The pharmacotherapy description listed several medications and their classes and explained the processes by which these medications affect neurotransmitters (e.g., reuptake inhibition):

“Antidepressant medications were discovered in the early 1950s and marketed shortly thereafter. A variety of antidepressant medications have been approved by the FDA to treat depression. In various ways, different antidepressants seem to affect how neurotransmitters behave. Neurotransmitters are chemicals in the brain which pass signals from one neuron (brain cell) to another. They may activate or inhibit a signal to the next neuron, allowing messages to be sent along or suppressed. Neurotransmitters play a role in a wide variety of brain functions, including the experience of emotions, motivation, and energy level. Though the biology of why antidepressants work is not completely understood, they appear to affect how certain neurotransmitters, such as serotonin, dopamine, and norepinephrine, are treated in the brain.

What are antidepressant medications? Antidepressants comprise a variety of different oral medications, including reuptake inhibitors, tricyclic antidepressants, monoamine oxidase inhibitors, and others. Here we will review just one type of antidepressant medication – reuptake inhibitors.

Reuptake is the process in which neurotransmitters are naturally reabsorbed back into the neuron that released it. A reuptake inhibitor prevents this from happening. Instead of getting reabsorbed, the neurotransmitter stays, at least temporarily, in the gap between the neurons, called the synapse. There are three main classes of reuptake inhibitors which target different transmitters. Selective serotonin reuptake inhibitors (SSRIs), such as fluoxetine (Prozac) and sertraline HCL (Zoloft), are the most commonly prescribed antidepressants available. Serotonin and norepinephrine reuptake inhibitors (SNRIs), such as duloxetine HCL (Cymbalta) and venlafaxine HCL (Effexor), are among the newer types of antidepressants. Norepinephrine and dopamine reuptake inhibitors (NDRIs) are the third class, but they are represented by only one drug: buproprion HCL (Wellbutrin).

What does the patient experience? In general, a patient must take regular doses of a prescribed antidepressant for several weeks before they are likely to have the medication’s full effect. Medication should not be discontinued without speaking with the doctor, even if the patient feels better, as stopping can result in discontinuation syndrome (see next section), or depression could return. A significant percentage of people may not respond to a prescribed antidepressant. In these cases, switching to a different medication or adding another medication can sometimes help treat symptoms.

What are the possible side effects/risks? Common side effects of antidepressants can include nausea and vomiting, weight gain, diarrhea, sleep disturbances, and sexual problems. Changes in appetite or weight are largely drug-dependent and are related to which neurotransmitters they affect. Tricyclics, for example, have the effect of weight gain and/or increased appetite, whereas buproprion and venlafaxine achieve the opposite effect. Sexual side-effects, such as loss of sexual drive and erectile dysfunction, are common with SSRIs. Although usually reversible with discontinuation of the medicine, these sexual side-effects can, in rare cases, last for months or years after the drug has been completely withdrawn.

Some antidepressants can have serious risks. Studies have shown that the use of antidepressants is associated with an increased risk of suicidal behavior and thinking in those under 25. Some antidepressant medications might harm a fetus if taken during pregnancy. Patients taking monoamine oxidase inhibitors must avoid certain foods that contain high levels of the chemical tyramine. This chemical is in some medications and in many cheeses, wines, and pickles. Consuming this chemical while taking a monoamine oxidase inhibitor may cause a sharp increase in blood pressure, which could lead to a stroke or other complications.

Other risks of antidepressant medications include serotonin toxicity and discontinuation symptoms. Serotonin toxicity is an excess of serotonin that can induce mania, restlessness, agitation, emotional lability, insomnia, and confusion. Although this condition is serious, it is relatively rare, generally only appearing at high doses or while on other medications. When proper medical intervention has been taken within about 24 hours, this syndrome is rarely fatal. Discontinuation symptoms may occur once the patient has taken the antidepressant for at least four weeks and then abruptly discontinues the medication. Common symptoms include flu-like symptoms, sleep disturbances, sensory/movement disturbances, mood disturbances, and cognitive disturbances. Most cases of discontinuation syndrome last between one and four weeks, are relatively mild, and resolve on their own.”

***Talk Therapy Description***

The talk therapy description focused on describing cognitive behavioral therapy:

**“Psychotherapy, or talk therapy, has been used to treat major depression since the late 19^th^ century, and it has been studied since the mid 20^th^ century. One form of talk therapy for depression which has been studied extensively is cognitive behavioral therapy (CBT). CBT therapists help patients to identify and change their negative thinking patterns and problematic behaviors. CBT is guided by the principle that our beliefs powerfully shape our emotions and behavior. Though the exact mechanisms of why CBT works are not completely understood, the therapy appears to affect the ways people think about themselves, the world around them, and their future.**

**What is CBT? CBT involves the patient talking with a therapist or counselor to better understand their difficulties and to learn coping skills to manage their depression.**

**CBT involves four phases of treatment. In the initial stage, the therapist provides educational information to the patient about the nature and causes of the mental disorder. The therapist explains the role of negative thinking patterns and problematic behaviors in maintaining the disorder. In the second stage, the therapist guides the patient in learning to identify the negative thoughts that arise during emotional situations and how to recognize the cognitive biases, or logical errors, in these thinking patterns. The therapist teaches the patient how to challenge these negative thoughts to arrive at more objective, less negative conclusions, a process called cognitive restructuring. In the third stage, the therapist helps the patient to change behaviors that contribute to depression, such as isolation and withdrawing from challenges. During this phase, the patient practices their cognitive restructuring skills to ease the difficulty of changing behaviors. In the final phase of therapy, the therapist helps the patient to set goals and plan for what to do if depression returns.**

**What does the patient experience?** CBT involves the patient attending weekly therapy sessions with the therapist. CBT can be conducted individually, wherein the patient meets alone with the therapist each week, or it can be conducted in group format, wherein the same group of patients meets weekly with one or two therapists who lead the group. Therapy sessions may occur in a variety of settings, including private practice offices, hospitals, specialty clinics, and community mental health centers. Occasionally, therapists conduct internet-based CBT. A typical session lasts 50 to 90 minutes. Typically, patients attend therapy for 16-30 sessions (or approximately 4 to 8 months).

**What are the possible side effects/risks?  In general, there’s little risk in receiving CBT. Because it can explore painful feelings and experiences, patients may feel emotionally uncomfortable at times. Patients may cry, get upset, feel angry, or feel physically drained during a challenging therapy session. Some forms of CBT may encourage patients to confront situations they would rather avoid – such as practicing assertiveness at work. This can lead to temporary stress or anxiety. Talk therapy may also result in changes that were not originally intended (such as divorce or remaining in a relationship the patient believed they would leave). For people in some professions (e.g., politics, law enforcement), the fact of being in therapy, if it becomes public, may negatively affect their career. There is a small risk that the patient’s condition will worsen due to treatment.”**

**Depression and Anxiety Symptoms**

Participants completed the 8-item Patient-Reported Outcomes Measurement Information System – Anxiety scale (PROMIS-A) and the 8-item Depression scale (PROMIS-D). (1, 2). These scales assess anxiety and depression over the past seven days, with items such as “My worries overwhelmed me” (PROMIS-A) and “I felt depressed” (PROMIS-D), rated on a 5-point Likert-type scale from “Never” to “Always.” Items were summed and converted to *T*-scores using conversion tables (for short forms 8a; https://www.assessmentcenter.net/). Internal consistency for both scales was excellent in the current sample (PROMIS-A *α* = .92; PROMIS-D Cronbach’s *α* = .92).

**Study 3 Supplemental Results**

**Sample Description: Mental Health Experience**

The proportions of participants who reported personal experience with mental illness in the current study were similar to the proportions in Studies 1 and 2. In the current sample, just over half of participants (51.0%) reported that they or a close loved one had ever been diagnosed with a mental illness, just less than one third reported that they had not (29.9%), and the remaining participants reported “Unsure” or did not respond (19.2%). Likewise, with regard to whether the participant or a close loved one had ever been treated for a mental illness, just under half reported “Yes” (45.5%), over one third reported “No” (39.0%), and the remaining participants reported “Unsure” or did not respond (15.6%).

Mean *T*-scores on the brief self-report anxiety and depression scales were slightly higher in the current sample than the standardized sample average (anxiety *T*-score *M* = 56.9, *SD* = 9.0; depression *T*-score *M* = 54.2, *SD* = 8.2).

**Preliminary Analyses: Checking for Failure of Random Assignment**

To check for failure of random assignment, we first examined whether the four depression description conditions differed on age, gender, or race/ethnicity. A univariate ANOVA on age was non-significant, indicating the four depression conditions did not differ on age, *F*(3, 299) = 0.15, *p* = .93, ${}_{p}^{2}$ = .001. Chi-square tests likewise revealed that the conditions did not differ on gender (men vs. women), ${}^{2}$(3, *N* = 306) = 1.57, *p* = .67, *φ* = .07, or on race/ethnicity (Hispanic/Latinx vs. Asian-American vs. Black/African-American vs. White/Caucasian vs. all other), ${}^{2}$(12, *N* = 308) = 12.82, *p* = .38, Cramer’s *V* = .12.

**Primary Analyses: Effect of Depression Description on Perceptions of rTMS**

We compared the four conditions on each of the four rTMS perception items (*likelihood of positive effects*, *likelihood of negative effects*, *likelihood of pursuing*, and *likelihood of recommending*) using univariate ANOVAs and follow-up independent samples *t*-tests as warranted.

The omnibus ANOVAs for both likelihood of pursuing rTMS and likelihood of recommending rTMS approached statistical significance [pursuing rTMS: *F*(3, 304) = 2.41, *p* = .067, ${}_{p}^{2}$ = .02; recommending rTMS: *F*(3, 304) = 2.47, *p* = .062, ${}_{p}^{2}$ = .02]. Follow-up pairwise comparisons revealed that the brain circuitry-based description yielded significantly better perceptions of rTMS than the psychologically-based description [pursuing rTMS: *t*(152) = 2.71, *p* = .007, Cohen’s *d* = 0.44; recommending rTMS: *t*(152) = 2.71, *p* = .008, Cohen’s *d* = 0.44). Two other tests approached statistical significance, the first with the control condition endorsing marginally higher likelihood of pursuing rTMS compared to the psychologically-based description, *t*(151) = 1.84, *p* = .067, Cohen’s *d* = 0.30, and the second with the brain circuitry-based description yielding marginally higher likelihood of recommending rTMS compared to the neurotransmitter-based description, *t*(153) = 1.80, *p* = .07, Cohen’s *d* = 0.29. No other pairwise comparison on these two dependent variables approached statistical significance, *p*s > .13. Likewise, omnibus ANOVAs comparing the four conditions on the remaining two dependent variables (likelihood of positive and negative effects of rTMS) were not significant, *p*s > .21.

**Secondary Analyses: Gender Moderation**

We next explored whether gender (men versus women) interacted with condition to predict the four rTMS perception items. Data for the two participants who identified as “other” gender were dropped from these analyses. Results of the 2 gender x 4 condition ANOVAs revealed that gender interacted with condition for two dependent variables, namely, for likelihood of pursuing rTMS, *F*(3, 298) = 3.34, *p* = .02, ${}_{p}^{2}$ = .03, and for likelihood of recommending rTMS, *F*(3, 298) = 3.40, *p* = .02, ${}_{p}^{2}$ = .03. Follow-up independent samples *t*-tests for pairwise comparisons of the four conditions within each gender revealed that the pattern of results in women was partially consistent with our original set of hypotheses. Among women, rTMS was perceived more positively (both higher likelihood of pursuing and recommending rTMS) following the brain-circuitry-based causal description compared to each of the other three descriptions, and the remaining three descriptions did not result in different perceptions of rTMS. See Table S2 for full statistical results of these pairwise comparisons. Among men, the only differences that emerged were such that the psychologically-based description resulted in significantly lower likelihood of pursuing rTMS than either the control or neurotransmitter-based descriptions and lower likelihood of recommending rTMS than the control condition. In sum, it appears that the brain circuitry-based description had a unique positive impact on the perceptions of rTMS in the women in our study, whereas the psychologically-based description had a somewhat negative impact on the perceptions of rTMS in men in our study.

**Table S2**

Pairwise Comparisons of Likelihood of Pursuing and Recommending rTMS by Condition within Men and Women

|  | **Men** |  |  | **Women** | | | | | |  |
| --- | --- | --- | --- | --- | --- | --- | --- | --- | --- | --- |
|  | df | *t* | Cohen’s *d* | df | | *t* | | Cohen’s *d* | |  |
| **Likelihood of pursuing rTMS** | | | | |  | |  | |  | |
| BC vs NT | 65 | 1.48 | 0.37 | 85 | | 2.48* | | 0.54 | |  |
| BC vs PSY | 70 | 0.80 | 0.19 | 79 | | 2.46* | | 0.55 | |  |
| BC vs CON | 65 | 1.48 | 0.37 | 85 | | 2.48* | | 0.54 | |  |
| NT vs PSY | 72 | 2.02* | 0.48 | 76 | | 0.43 | | 0.10 | |  |
| NT vs CON | 67 | 0.34 | 0.08 | 82 | | 0.16 | | 0.04 | |  |
| PSY vs CON | 71 | 2.33* | 0.55 | 76 | | 0.19 | | 0.04 | |  |
| **Likelihood of recommending rTMS** | | | | |  | |  | |  | |
| BC vs NT | 65 | 1.25 | 0.31 | 85 | | 3.12** | | 0.68 | |  |
| BC vs PSY | 70 | 0.73 | 0.17 | 79 | | 2.57* | | 0.58 | |  |
| BC vs CON | 65 | 1.25 | 0.31 | 85 | | 3.12** | | 0.68 | |  |
| NT vs PSY | 72 | 1.47 | 0.35 | 76 | | 0.16 | | 0.04 | |  |
| NT vs CON | 67 | 0.59 | 0.14 | 82 | | 0.08 | | 0.02 | |  |
| PSY vs CON | 71 | 2.02* | 0.48 | 76 | | 0.23 | | 0.05 | |  |

*Note*. BC = Brain circuitry-based causal description; NT = Neurotransmitter-based causal description; PSY = Psychologically-based causal description; CON = Control description. **p* < .05, ***p* < .01

**Secondary Analyses: Exploration of rTMS Ratings Across All Three Studies**

Given that Studies 1 and 2 were conducted in MTurk samples, whereas Study 3 was conducted in an undergraduate sample, it could be argued that familiarity and perceptions of rTMS may differ in important ways across these differing sample demographics, so results of Studies 1 and 2 may not be consistent in the Study 3 sample. To examine this question, we compared *familiarity* and perceptions of (i.e., *likelihood of positive effects*, *likelihood of negative effects*, *likelihood of pursuing*, and *likelihood of recommending*) rTMS across the three studies, in Study 3 averaging across the four depression description conditions. As depicted in Figure S1, familiarity of rTMS was slightly lower in Study 1 than in Study 3, *t*(413) = 3.03, *p* = .003, Cohen’s *d* = 0.34, but familiarity of rTMS did not differ between Study 2 and Study 3, *t*(412) = 0.78, *p* = .44, Cohen’s *d* = 0.09. Identical to the pattern of findings of familiarity of rTMS found in Study 1, in Study 3, rTMS was rated as less familiar than either pharmacotherapy, *t*(307) = 13.25, *p* < .001, Cohen’s *d* = .76, or talk therapy, *t*(307) = 26.90, *p* < .001, Cohen’s *d* = 1.53.

Perceptions of rTMS were also in a similar range in Study 3 as compared to Studies 1 and 2. Perceptions of rTMS were slightly better in Study 3 than in Study 1, *p*s < .01, Cohen’s *d*s = 0.29 – 0.49, consistent with the results from Study 2 which revealed that the longer rTMS description yielded better perceptions than the shorter description (from Study 1). Perceptions of rTMS were slightly worse in Study 3 than in Study 2, *p*s < .016, Cohen’s *d*s = 0.28 – 0.33. This pattern of results indicates that rTMS was perceived relatively similarly in the undergraduate student sample as compared to the two MTurk samples.

**Figure S1**

*
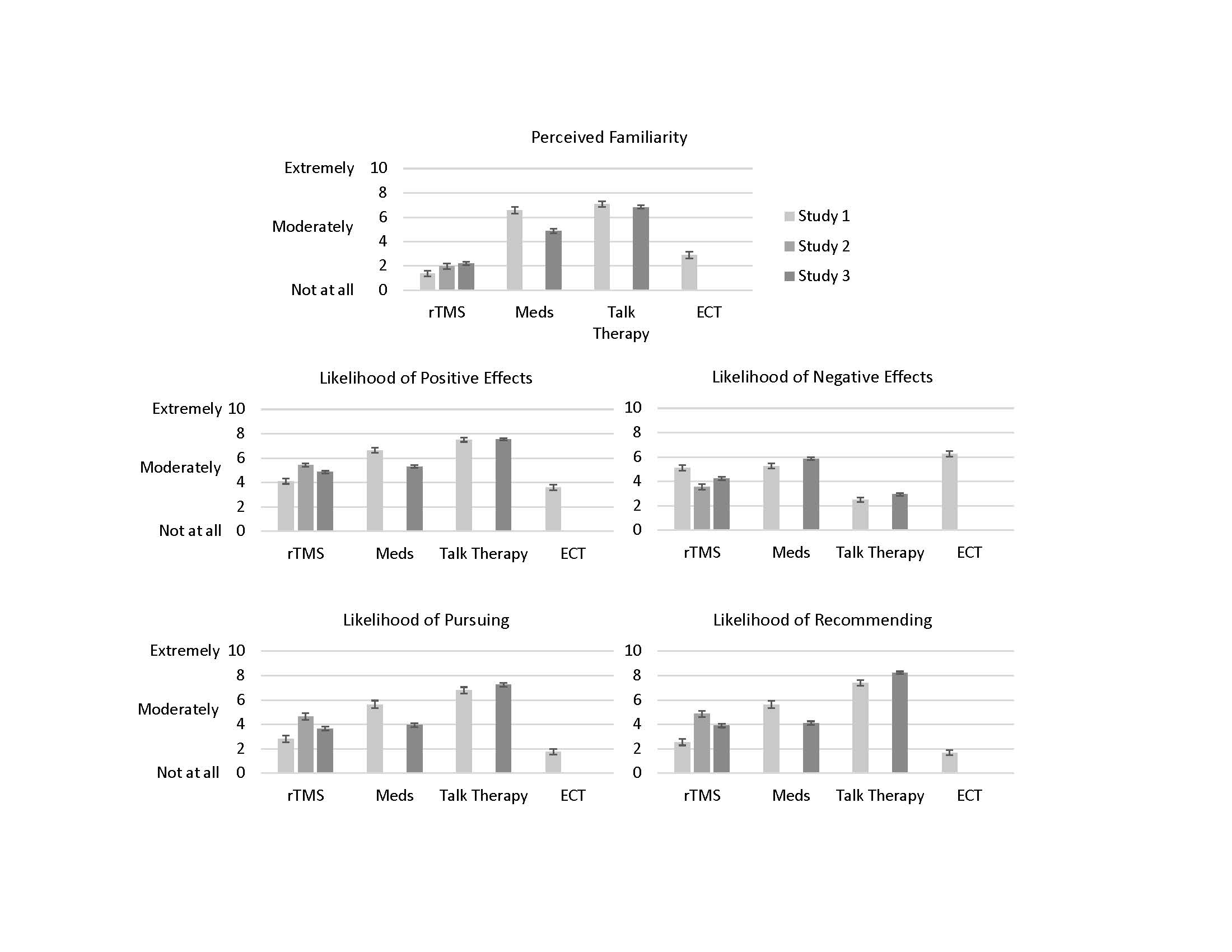
Perceived Familiarity and Perceptions of rTMS and Other Treatments Across All Three Studies*

*Note.* This figure depicts mean ratings on the perceived familiarity and four treatment perception items in Studies 1 (*N* = 107; MTurk), 2 (*N* = 106; MTurk), and 3 (*N* = 308; undergraduates). In Study 1, brief descriptions of each treatment were displayed to participants prior to each treatment being rated. In Study 2, a long description of rTMS was displayed to participants prior to it being rated (no other treatments were assessed). In Study 3, participants were randomly assigned to read one of four descriptions of depression after which a long description of each treatment was displayed to participants. Ratings of each treatment were made immediately after each treatment description was displayed. Error bars are standard errors. Meds = Pharmacotherapy**.**

# References

**References**

1. Cella D, Riley W, Stone A, Rothrock N, Reeve B, Yount S, *et al.* (2010): The Patient-Reported Outcomes Measurement Information System (PROMIS) developed and tested its first wave of adult self-reported health outcome item banks: 2005–2008. *J Clin Epidemiol* 63: 1179-1194. doi:10.1016/j.jclinepi.2010.04.011
2. Pilkonis PA, Choi SW, Reise SP, Stover AM, Riley WT, Cella D, *et al.* (2011): Item banks for measuring emotional distress from the Patient-Reported Outcomes Measurement Information System (PROMIS®): depression, anxiety, and anger. *Assessment* 18: 263-283. doi:10.1177/1073191111411667
